# Sexual Satisfaction Among Sexual Minority and Heterosexual Middle-Aged and Older Adults

**DOI:** 10.1093/geroni/igad010

**Published:** 2023-01-31

**Authors:** Elżbieta W Buczak-Stec, Hans-Helmut König, André Hajek

**Affiliations:** Department of Health Economics and Health Services Research, University Medical Center Hamburg-Eppendorf; Hamburg Center for Health Economics, Hamburg, Germany; Department of Health Economics and Health Services Research, University Medical Center Hamburg-Eppendorf; Hamburg Center for Health Economics, Hamburg, Germany; Department of Health Economics and Health Services Research, University Medical Center Hamburg-Eppendorf; Hamburg Center for Health Economics, Hamburg, Germany

**Keywords:** Health status, LGB, LGBT, Loneliness, Minority stress, Older adults, Partnership satisfaction, Satisfaction with sex life, Sexual orientation, Sexual minority adults, Sexual satisfaction, Sexuality, Satisfaction, Social isolation

## Abstract

**Background and Objectives:**

Sexual satisfaction is an important part of sexual health and overall well-being. A large number of older people continue to be sexually active, and many are satisfied with their sex life. However, little is known about whether sexual satisfaction differs according to sexual orientation. Therefore, the aim of the study was to investigate whether sexual satisfaction differs according to sexual orientation in later life.

**Research Design and Methods:**

The German Ageing Survey is a nationally representative study of the German population aged 40+. In the third wave (2008), data on both sexual orientation (heterosexual, homosexual, bisexual, other) and sexual satisfaction (1—very dissatisfied to 5—very satisfied) were collected. Multiple regression analyses with sampling weights were performed (stratified by age: 40–64; 65+).

**Results:**

We included 4,856 individuals in our analysis (mean age 57.6 ± 11.6; 40–85 years, 50.4% were women, 92.3% (*n* = 4,483) were heterosexual, and 7.7% (*n* = 373) were sexual minority adults). In sum, 55.9% of heterosexual individuals and 52.3% of sexual minority adults were satisfied or very satisfied with their sex life. Multiple regression analysis showed that sexual orientation was not significantly associated with sexual satisfaction among both middle-aged (β = 0.07; *p* = .45) and older adults (β = 0.01; *p* = .87). Higher sexual satisfaction was associated with lower loneliness scores, partnership satisfaction, importance of sexuality and intimacy, and better health status.

**Discussion and Implications:**

Our analysis showed that sexual orientation was not significantly associated with sexual satisfaction among both middle-aged and older adults. Lower loneliness, better health status, and partnership satisfaction significantly contributed to higher sexual satisfaction. Approximately 45% of older individuals (aged 65 years and older), regardless of their sexual orientation, were still satisfied with their sex life.

**Translational Significance:** The field of sexual satisfaction *in late life* as related to sexual orientation is a neglected area in research. We reported the level of sexual satisfaction with regard to sexual orientation and age and showed that satisfaction with sex life declined in a similar way with age among both heterosexual and sexual minority adults. We showed that sexual orientation was not significantly associated with sexual satisfaction. Lower loneliness, better health status, and partnership satisfaction significantly contributed to higher sexual satisfaction.

## Background and Objectives

Sexual satisfaction is recognized as an important part of sexual health and overall well-being for both middle-aged and older individuals. Research concerning sexual satisfaction among older individuals is gaining attention (Buczak-Stec, König, & Hajek, 2021; Stentagg et al., 2021; Vasconcelos et al., 2021). Individuals in older age are engaging in sexual activity (Lindau et al., 2007), and are satisfied with their sex life (Stentagg et al., 2021). Sexual satisfaction, also in older age, is not restricted to intercourse. It encompasses satisfaction with all kinds of sexual activities that an individual values, for example, intercourse, kissing, fondling, or masturbation. Experiencing and expressing sexuality comes through thoughts, behaviors, and practices (World Health Organization, 2017). Sexual activity and sexual satisfaction are important for both partnered and nonpartnered individuals (DeLamater, 2012).

### Sexual Orientation and Sexual Satisfaction

Although research on sexual well-being and sexual satisfaction has focused predominantly on relatively young heterosexual individuals, often in the context of marriage (Pronier & Monk-Turner, 2014), experiencing and expressing sexuality are relevant for all adults, regardless of age and sexual orientation (Björkenstam et al., 2020; Lee et al., 2016). Investigating sexual satisfaction in older individuals is important, as it has been suggested that sexual interest and sexual satisfaction may contribute to successful aging (Buczak-Stec et al., 2019; Štulhofer et al., 2019), support well-being (Lyons et al., 2013), and also increase the overall quality of life (Chao et al., 2011).

As the population of older individuals grows, the number of sexual minority older adults is also growing (Buczak-Stec, König, Riedel-Heller, & Hajek, 2021). Research pertaining to differences in sexual satisfaction with regard to sexual orientation among older individuals is scarce. According to a recent systematic review, only a few studies on sexual satisfaction included information about sexual orientation (Rausch & Rettenberger, 2021). Although more studies have been published in recent years, the results are still inconclusive. Some evidence suggests similarities in the level of sexual satisfaction among heterosexual and sexual minority adults (Del Mar Sánchez-Fuentes & Sierra, 2015; Kuyper & Vanwesenbeeck, 2011). On the other hand, significant differences in sexual satisfaction with regard to sexual orientation were reported in recent large studies, for example, in England, Sweden, and the United States (Björkenstam et al., 2020; Flynn et al., 2017; Grabovac et al., 2019).

As age progresses, the probable differences in sexual satisfaction between heterosexual and sexual minority adults may become more pronounced. There are several possible explanations for these potential differences. Numerous studies have shown that sexual minority adults are at greater risk of poorer health in comparison to heterosexual individuals, for example, due to long-lasting exposure to discrimination and prejudice (Meyer, 2003). Poor health is a known risk factor that contributes to sexual dissatisfaction (Buczak-Stec, König, & Hajek, 2021; Flynn et al., 2016; Lee et al., 2016). Sexual minority individuals in middle and older age also differ in terms of their partnership and family status. Sexual minority adults are more likely to live alone, not have a partner, or have a shorter length of their relationship than heterosexual individuals (Fokkema & Kuyper, 2009; Kim & Fredriksen-Goldsen, 2014). Partner availability, relationship satisfaction, and health status are significant factors influencing engagement in sexual activity in older age, which further contributes to sexual satisfaction (Buczak-Stec, König, & Hajek, 2021; Freak-Poli, 2020). In addition, it has been shown that experiencing discrimination, suppression, or stigmatization can have a negative impact on the relationships of sexual minority adults, which in turn may lead to lower sexual satisfaction (Ritter et al., 2018). The prevalence of other risk factors affecting sexual satisfaction, such as lack of social support, higher loneliness scores, and sexual violence, is higher among sexual minority adults (Björkenstam et al., 2020; Buczak-Stec, König, & Hajek, 2021; Buczak-Stec et al., 2023; Peterson et al., 2023).

Moreover, in numerous countries, for many years, it was difficult or almost impossible for sexual minority adults to express their sexuality in public. On the one hand, this long-lasting minority stress, stigmatization, and experiences of internalized homophobia may have an additional negative impact on the level of sexual satisfaction (Srinivasan et al., 2019). On the other hand, perhaps the changes in sexual norms in recent decades may have led to sexual minority adults being able to express their sexuality (e.g., holding hands, kissing) in public places, at least in certain countries, cities, or neighborhoods, which may have contributed to increased sexual satisfaction among sexual minority adults.

Another possible explanation of potential differences in sexual satisfaction with regard to sexual orientation is related to differences in sexual attitudes. It is possible that older heterosexual individuals have more conservative sexual attitudes than older sexual minority adults (Grollman, 2017). This, in turn, may indicate that their sexual satisfaction is lower in comparison to sexual minority adults. The combination of all these factors could lead to differences in sexual satisfaction between sexual minority and heterosexual middle-aged and older adults.

### Conceptual Framework of Sexual Satisfaction—Ecological Model

Factors that influence satisfaction with sex life have been explored in several studies; however, these studies are predominantly exclusively based on heterosexual samples. The determinants of sexual satisfaction are often structured at the individual, relationship, and society/cultural levels (Carpenter et al., 2009). Important insights into the conceptualization of sexual satisfaction were also gained by Del Mar Sánchez-Fuentes et al. (2014). The authors summarized and structured the determinants of sexual satisfaction using the ecological model, dividing determinants into microsystem (e.g., age, sociodemographic variables, physical health, lifestyle-related variables, self-esteem), mesosystem (e.g. relationship satisfaction, marital status, sexual assertiveness), exosystem (e.g. social status, social support, discrimination), and macrosystem (e.g., religious beliefs). It has been shown that, among other things, self-rated health (Castellanos-Torres et al., 2013), multimorbidity (Appa et al., 2014), mental health (Heywood et al., 2018), functional status (Matthias et al., 1997), frequency of sexual intercourse (Heywood et al., 2018), interpersonal factors (Gillespie, 2017), marital status (McCall-Hosenfeld et al., 2008), relationship satisfaction (Del Mar Sanchez-Fuentes et al., 2016; Kim & Jeon, 2013), and partner’s health status (Fisher et al., 2015) are associated with sexual satisfaction.

In this proposed model (Del Mar Sánchez-Fuentes et al., 2014), sexual orientation is listed as a microsystem factor. However, to date, only a few studies have explored sexual satisfaction and its relation to sexual orientation. Existing studies have delivered contradictory results (Frederick et al., 2021; Kuyper & Vanwesenbeeck, 2011).

In sum, there is an incomplete understanding of the potential differences in sexual satisfaction according to sexual orientation. The field of sexual satisfaction *in late life* with regard to sexual orientation is a neglected area in research.

Drawing on the ecological model (Del Mar Sánchez-Fuentes et al., 2014), the aim of our study is to investigate, whether sexual satisfaction differs according to sexual orientation in later life. We will present our findings stratified by two age groups— middle-aged (40–64 years) and older individuals (aged 65 years and above).

Taking into account the conceptual framework of sexual satisfaction (Del Mar Sánchez-Fuentes et al., 2014), and recent research showing, among other things, worse health status, lower social support, higher loneliness scores, and higher likelihood of not having a partner among sexual minority older adults (Björkenstam et al., 2020; Buczak-Stec et al., 2023; Nelson & Andel, 2020; Seelman, 2019), we hypothesized that sexual minority adults have lower sexual satisfaction than their heterosexual counterparts.

## Research Design and Methods

### Study Design and Participants

To conduct this study, we used nationally representative data from the German Ageing Survey (DEAS). DEAS is a cross-sectional and longitudinal survey of community-dwelling individuals aged 40 years and older. The first-wave data were collected in 1996 (*n* = 4,838), the second wave took place 6 years later in 2002 (*n* = 3,670), and the third wave in 2008. The subsequent waves took place at intervals of 3 years (2011, 2014, and 2017). Eligible participants were community-dwelling individuals who lived in Germany and were at least 40 years old. Participants consist of random samples drawn from population registers (stratified to age group, sex, and part of the country [West Germany/East Germany]). A two-stage sampling procedure was used for the samples. First, from a total of 12,000 municipalities that existed in Germany at that time, a random sample of 290 municipalities was drawn in 1996. The local population registries of these 290 municipalities served as the foundation for sampling the population of individuals living in private households in the community and ranging in age from 40 to 85 years old (Klaus et al., 2017). The methods of data collection in the DEAS survey include a personal interview (at participant’s home) and a self-completed questionnaire (“drop-off”). Questions pertaining to private, personal, or intimate data such as sexual orientation, income, and satisfaction with sex life are incorporated in the drop-off questionnaire. Further details on the study design and methods can be found in Klaus et al. (2017). The drop-off questionnaire was filled-in by 74.3% of the individuals. Further, out of eligible respondents, 91.6% provided the answer concerning sexual satisfaction and 87.6% provided an answer about sexual orientation. Participants who provided this information were younger, had a higher level of education, and a higher physical functioning.

Both questions on sexual orientation and sexual satisfaction were only included in the third wave. For this reason, we used cross-sectional data from the third wave (2008) in our study. It consists of the panel participants (from Wave 1 and Wave 2; *n* = 1.991) and additional, newly drawn individuals aged 40–85 years (*n* = 6.205).

All DEAS procedures were performed in accordance with the ethical standards of the institutional and/or national research committee and with the 1964 Helsinki declaration and its later amendments or comparable ethical standards. Permission from institutional review boards or ethics committees was not necessary, as the criteria for requiring an ethics statement were not met (e.g., risk for the respondents, lack of information about the aims of the study, examination of patients). Participants provided informed written consent to take part in the study.

### Dependent Variable

Our outcome variable was satisfaction with sex life. In order to measure sexual satisfaction, participants were asked the following question: “How satisfied are you with your sex life?”. There were five response options: 1 = very dissatisfied, 2 = dissatisfied, 3 = neither satisfied nor dissatisfied, 4 = satisfied, and 5 = very satisfied.

### Independent Variables

Informed by the ecological model proposed by Del Mar Sánchez-Fuentes et al. (2014), determinants of sexual satisfaction were chosen. Based on this model, we segregated the variables into the (a) individual level (microsystem), (b) partnership level (mesosystem), (c) social support variables (exosystem), and (d) macrolevel (macrosystem).

On the *individual level* (*microsystem*), sexual orientation was our main variable of interest. In the self-administrated questionnaire, respondents were asked “How would you describe your sexual orientation?”, with four possible answers: heterosexual, homosexual, bisexual, and other. In accordance with previous research, in our analysis, we categorized sexual orientation into two groups: heterosexual and sexual minority (homosexual, bisexual, and others).

Variables on individual level were divided into sociodemographic factors, lifestyle-related variables, and possibility of sexual activity (e.g., health status). Namely, we adjusted for sex, age, and labor force participation (working, retired, and other: not employed). Educational attainment was determined according to the Internationally Standard Classification of Education ISCED-97 (low, medium, and high; Matthews et al., 2017). We also adjusted for type of district (large cities, urban cities, urban–rural districts, rural districts; *Indicators and maps for spatial and urban development [Indikatoren und Karten zur Raum- und Stadtentwicklung]*, XXXX). We included smoking status (never, used to, stopped smoking within the last year, occasionally, daily) and the frequency of physical activity (daily, several times a week, once a week, 1–3 times per month, less often, never). Furthermore, in our analysis, we incorporated a set of measures of physical health. A single self-rated health item was used to assess global health (1 = very good to 5 = very poor). We used the subscale “Physical Functioning” of SF-36 to measure physical functioning (Ware Jr & Sherbourne, 1992). It ranged from 0 to 100, with higher values representing better physical functioning. In our study, Cronbach’s alpha was 0.92. Depressive symptoms were measured with Center for Epidemiological Studies—Depression scale (CES-D; 0–45, higher values indicating more depressive symptoms; Radloff, 1977). In our study, Cronbach’s alpha was 0.87. We also adjusted for the total number of physical illnesses (sum score ranging from 0 to 11; e.g., cardiac disorders, diabetes, asthma). Self-esteem was assessed using a 10-item scale ranging from 1 to 4, with high values representing high self-esteem (established Rosenberg scale; in our study, Cronbach’s alpha was 0.82; Ferring & Filipp, 1996). We included the variable indicating whether sexuality and intimacy are important to the respondents. Individuals were asked “How much currently do you think of or do something about intimacy and sexuality?” (ranging from 1 = don’t think a lot about it, don’t do anything for it to 7 = think a lot about it, do a lot for it).

Variables on *partnership level* (*mesosystem*) pertained to the existence of partner or family status (married/partnership, divorced, widow, single), the overall assessment of the current relationship (for partnered individuals) and the assessment of relationship with own family. Partnered responders were asked “How would you rate your current relationship overall?” ranging from 1 = very good to 5 = very bad. Assessment of relationship with own family was measured on a 5-point Likert scale (1 = very good to 5 = very bad). In the group of *social support variables* (*exosystem*), we included social network size and loneliness. Social support was quantified with the following question: Respondents were asked to name the individuals who are most important to them and who they maintain regular contact with (individuals can include coworkers, neighbors, friends, acquaintances, relatives, and members of your household). The network size ranged from 0 to 9. Loneliness was measured with the de Jong-Gierveld, 6-item Scale for Loneliness (ranging from 1 to 4, where high values indicate great loneliness; in our study, Cronbach’s alpha was .83; de Jong-Gierveld & Kamphuls, 1985). On the *macrolevel* (*macrosystem*), we included religion/spirituality measures according to the frequency of going to church, the mosque, synagogue, or other religious assemblies (once or more per week; 1–3 times per month or several times a year; less often or never).

### Statistical Analysis

The link between sexual orientation and sexual satisfaction was investigated using multivariate linear regression models using robust standard errors. To account for non-answer to drop-off questionnaire, selective panel mortality, and in order to obtain a nationally representative sample, we used sampling weights. Analysis of variance was used to compare continuous variables, and Chi-square was used to compare categorical variables. Analyses were stratified by age into middle-aged (40–64 years old) and older individuals (aged 65 years and above). In sensitivity analysis, the missing values were also addressed using the full-information maximum-likelihood method (FIML; Enders & Bandalos, 2001). Analyses were performed using Stata 16.0. Statistical significance was set at *p* < .05.

## Results

### Study Design and Participants

The study sample comprised 4,856 individuals who provided information on all variables, 50.4% were female, mean age was 57.6 years (*SD* 11.6, range 40–85 years), 92.3% were heterosexual individuals (*n* = 4,483), and 7.7% identified as a sexual minority (*n* = 373). In the total sample, 55.7% of the individuals were satisfied or very satisfied with their sex life (60.1% of middle-aged individuals and 45.6% of older individuals; *p* < .001).

Sexual minority adults were older (60.7 years [*SD* 11.9] vs 57.4 years [*SD* 11.5]; *p* < .001), had a higher average number of chronic diseases (2.2 [*SD* 1.9] vs 1.9 [*SD* 1.7]; *p* < .001), had a smaller network size (4.4 [*SD* 2.8] vs 4.7 [SD 2.8]; *p* < .05), were lonelier (1.9 [*SD* 0.6] vs 1.8 [*SD* 0.6]; *p* < .001), and rated their partnership poorer than heterosexual adults (1.8 [*SD* 0.7] vs 1.7 [*SD* 0.7]; *p* < .05). Moreover, they differed in education, district they lived in, employment status, and family status (Table 1). In contrast, sexual minority and heterosexual adults did not differ with regard to the level of sexual satisfaction (3.4 [1.0]) vs 3.5 [1.0]; *p* = .45).

**Table 1.** Sample Characteristics of Respondents of DEAS Survey (third wave, 2008)

Variable	Total sample (*N* = 4,856)	Heterosexual adults (*n* = 4,483; 92.3%)	Sexual minority adults (*n* = 373; 7.7%)	*p* Value
	% or mean (*SD*)	% or mean (*SD*)	% or mean (*SD*)	
Individual level				
Age	57.60 (11.57)	57.36 (11.51)	60.71 (11.91)	<.001
Sex				.16
Male	49.6%	49.4%	52.2%	
Female	50.4%	50.6%	47.8%	
Employment status				<.001
Working	51.3%	52.3%	38.3%	
Retired	34.5%	33.5%	47.7%	
Other: not employed	14.2%	14.2%	14.0%	
Education level^a^				<.001
Low	10.4%	10.1%	14.2%	
Medium	54.1%	53.5%	61.1%	
High	35.5%	36.4%	24.8%	
Monthly income^b^	1,778.32 (1,571.46)	1,789.30 (1,606.17)	1,642.86 (1,044.88)	=.10
Type of district^c^				<.001
Large city	23.6%	22.8%	33.6%	
Urban city	41.6%	42.2%	34.4%	
Urban–rural district	20.3%	20.2%	21.0%	
Rural district	14.6%	14.8%	11.0%	
Church visits				.43
Several times a week or once a week	9.0%	9.0%	9.7%	
1–3 times per month/several times a year	28.3%	28.5%	25.9%	
Less often/never	62.7%	62.6%	64.4%	
Smoking habits				.01
Never	46.6%	45.9%	55.1%	
Used to	29.7%	30.3%	22.4%	
Stopped smoking	1.3%	1.2%	2.7%	
Occasionally	4.7%	4.7%	4.0%	
Daily	17.7%	17.9%	15.8%	
Physical activity				.03
Daily	7.1%	7.0%	7.7%	
Several times a week	25.2%	25.7%	18.6%	
Once a week	19.3%	19.4%	18.7%	
Between 1 and 3 times per month	7.5%	7.6%	7.0%	
Less often	12.5%	12.6%	11.0%	
Never	28.4%	27.7%	36.9%	
Self-rated health (range: 1 = very good to 5 = very bad)	2.35 (0.85); 1–5	2.35 (0.85); 1–5	2.39 (0.83); 1–5	.43
Number of physical illnesses (range: 0–10)	1.95 (1.69); 0–10	1.93 (1.67); 0–10	2.21 (1.93); 0–10	<.001
Physical functioning^d^	87.92 (19.42); 0–100	88.09 (19.33); 0–100	85.70 (20.51); 0–100	<.05
Depressive symptoms^e^	5.91 (5.85); 0–42	5.90 (5.84); 0–42	6.07 (6.04); 0–30	.60
Cognition^f^	46.54 (13.99); 5–92	46.73 (13.96); 5–92	44.13 (14.09); 8–92	<.001
Self-esteem^g^	3.41 (0.40); 2–4	3.41 (0.39); 2–4	3.31 (0.48); 2–4	<.001
Importance of sexuality and intimacy (from 1 = lowest to 7 = highest)	4.77 (1.67); 1–7	4.79 (1.66); 1–7	4.59 (1.71); 1–7	<.05
Partnership level				
Type of partnership				<.001
Married	73.9%	74.8%	61.5%	
Divorced	9.6%	9.5%	11.1%	
Widowed	8.3%	7.9%	13.3%	
Single	8.2%	7.8%	14.1%	
Overall assessment of current relationship (from 1 = very good to 5 = very bad)	1.65 (0.65); 1–5	1.65 (0.65); 1–5	1.75 (0.66); 1–5	<.05
Social support level				
Assessment of relationship with own family (from 1 = very good to 5 = very bad)	1.97 (0.80); 1–5	1.96 (0.78); 1–5	2.12 (0.97); 1–5	<.001
Number of important people in regular contact	4.7 (2.8)	4.7 (2.8)	4.4 (2.8)	<.05
Loneliness^h^ (from 1 = low to 4 = high)	1.8 (0.6)	1.8 (0.6)	1.9 (0.6)	<.001
Outcome measure				
Satisfaction with sex life (from 1 = very unsatisfied to 5 = very satisfied)	3.44 (0.99); 1–5	3.45 (0.99); 1–5	3.40 (1.01); 1–5	.45

*Notes*: Total sample and data stratified by sexual orientation. Weighted counts, means, and percentages are presented for all variables; *p* values: chi-square (categorical variables) or analysis of variance (ANOVA; continuous variables). DEAS = The German Ageing Survey.

^a^ Education level according to ISCED (Matthews et al., 2017).

^b^ Income: monthly equivalence income (new OECD equivalence scale).

^c^ Type of district according to Federal Institute for Research on Building, Urban Affairs, and Spatial Development.

^d^ Physical functioning ranged from 0 = worst score to 100 = best score. It was measured by the subscale “Physical Functioning” of SF-36 Short Form Health Survey (Ware Jr & Sherbourne, 1992).

^e^ The Center for Epidemiological Studies—Depression scale (CES-D) was used to quantify depressive symptoms (Radloff, 1977).

^f^ Cognitive functioning was assessed with help of adaptation of the Digit Symbol Substitution Test; range 1–92, with higher values indicate better cognitive functioning.

^g^ Self-esteem was measured using the Rosenberg scale; range 1–4, with high values representing high self-esteem (Ferring & Filipp, 1996).

^h^ Loneliness was assessed using the De Jong Gierveld Loneliness scale.

**Figure 1.**
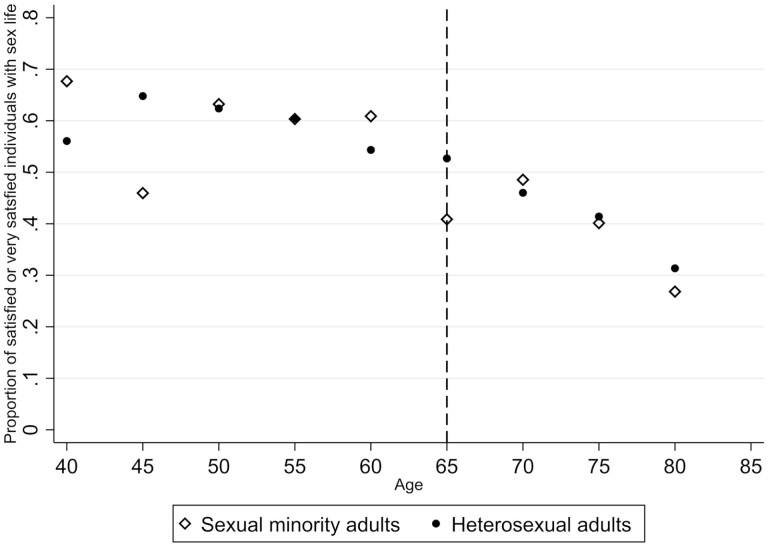
Proportion of satisfied or very satisfied individuals with sex life by age and sexual orientation.

In sum, 55.9% of heterosexual and 52.3% of sexual minority adults were satisfied or very satisfied with their sex life (*p* = .052). Further, satisfaction with sex life declined in a similar way with age among both heterosexual and sexual minority adults. About 61% of individuals aged 40–50 years were satisfied or very satisfied with their sex life. This number dropped to approximately 42% for individuals aged 70–80 years (Figure 1).

### Main Results

No significant association between sexual orientation and sexual satisfaction was identified in bivariate analysis (β = −0.049, *p* = .64 and β = 0.008, *p* = .90, respectively, for middle-aged and older individuals). Multiple regression analysis showed that, after adjustment of individual, partnership, social support, and macrolevel variables, sexual orientation was not significantly associated with sexual satisfaction among both middle-aged (β = 0.074, *p* = .45) and older adults (β = 0.012, *p* = 0.87; Table 2).

**Table 2.** Results of Multiple Regression Analysis

Variable	Total sample		Middle-aged adults (40–64 years)		Older adults (65 years and above)	
	Coef.	*SE*	Coef.	*SE*	Coef.	*SE*
Individual level						
Sexual orientation (ref. heterosexual) Sexual minority adults	0.057	(0.065)	0.074	(0.099)	0.012	(0.072)
Female (ref. male)	0.017	(0.037)	−0.019	(0.047)	0.073	(0.051)
Age	−0.001	(0.003)	−0.002	(0.004)	−0.001	(0.005)
Level of education (ref. low)						
Medium	−0.008	(0.066)	0.092	(0.113)	−0.094	(0.066)
High	−0.072	(0.071)	0.001	(0.116)	−0.114	(0.074)
Type of district (ref. large cities)						
Urban cities	0.025	(0.042)	−0.001	(0.057)	0.075	(0.054)
Urban–rural districts	0.124*	(0.049)	0.117	(0.065)	0.126*	(0.061)
Rural districts	0.045	(0.053)	0.029	(0.073)	0.069	(0.062)
Labor force status (ref. working)						
Retired	−0.035	(0.055)	−0.004	(0.073)	0.008	(0.187)
Not employed	−0.002	(0.057)	0.023	(0.063)	0.060	(0.205)
Physical functioning	−0.002*	(0.001)	−0.003	(0.002)	0.000	(0.001)
Total number of physical diseases	−0.032**	(0.011)	−0.040*	(0.016)	−0.019	(0.014)
Self-rated health	−0.042	(0.025)	−0.048	(0.033)	−0.022	(0.036)
Depressive symptoms	−0.011**	(0.004)	−0.012*	(0.005)	−0.007	(0.006)
Self-esteem	0.116*	(0.054)	0.155*	(0.069)	−0.004	(0.073)
Smoking habits (ref. never)						
Used to	−0.067	(0.037)	−0.061	(0.051)	−0.057	(0.049)
Stopped smoking	−0.365*	(0.180)	−0.296	(0.204)	−0.700*	(0.346)
Occasionally	−0.056	(0.095)	−0.044	(0.109)	−0.148	(0.196)
Daily	−0.111*	(0.054)	−0.116	(0.062)	−0.023	(0.094)
Physical activity (ref. daily)						
Daily	0.078	(0.075)	0.115	(0.100)	−0.019	(0.095)
Several times a week	0.013	(0.077)	0.015	(0.103)	−0.000	(0.099)
Once a week	0.051	(0.087)	0.110	(0.113)	−0.116	(0.119)
Between 1 and 3 times per month	0.085	(0.083)	0.095	(0.109)	0.076	(0.108)
Less often	0.056	(0.075)	0.043	(0.104)	0.079	(0.093)
Importance of sexuality and intimacy	0.108***	(0.012)	0.107***	(0.017)	0.115***	(0.015)
Partnership level						
Type of partnership (ref. married)						
Divorced	−0.095	(0.064)	−0.088	(0.076)	−0.090	(0.107)
Widowed	−0.038	(0.056)	−0.011	(0.107)	−0.037	(0.068)
Single	−0.277***	(0.070)	−0.350***	(0.080)	0.186	(0.115)
Social support variable						
Network size (number of important persons with whom you have with regular contact)	−0.008	(0.006)	−0.008	(0.008)	−0.004	(0.007)
Loneliness	−0.259***	(0.037)	−0.271***	(0.047)	−0.227***	(0.053)
Macrolevel						
Church visits (ref. several times a week or once a week)						
1–3 times per month/several times a year	−0.022	(0.061)	0.030	(0.092)	−0.103	(0.069)
Less often/never	−0.048	(0.059)	−0.018	(0.089)	−0.085	(0.068)
Constant	3.529***	(0.327)	3.480***	(0.423)	3.578***	(0.588)
Observations	4,856		2,767		2,089	
*R*-squared	0.126		0.136		0.123	

*Notes*: Sexual satisfaction and sexual orientation among middle-aged (40–64 years old) and older (65 years and above) individuals. Coef. = estimated coefficients; *SE* = robust standard errors in parentheses. Cross-sectional drop-off weights were included in regression models.

***p < .001. **p < .01. *p < .05.

Regression analysis revealed that among both middle-aged and older adults, thinking more of, or doing more about, intimacy and sexuality were positively associated with sexual satisfaction (β = 0.107, *p* < .001 and β = 0.115, *p* < .001, respectively). Additionally, lonelier individuals had a significantly lower satisfaction with sex life (β = −0.271, *p* < .001 and β = −0.227, *p* < .001, respectively). Among middle-aged individuals, but not older individuals, a lower number of physical diseases (β = −0.040, *p* < .05), less depressive symptoms (β = −0.012, *p* < .001), and higher self-esteem (β = 0.155, *p* < .05) were positively associated with higher satisfaction with sex life. Among other things, age, gender, education, labor force participation, self-rated health, and frequency of going to church were not significantly associated with sexual satisfaction in both age groups.

In sensitivity analysis, we estimated the models separately for heterosexual and sexual minority adults (Supplementary Table 1). The results showed that for both heterosexual and sexual minority adults, higher sexual satisfaction was associated with lower loneliness scores (β = −0.250, *p* < .001; β = −0.358, *p* < .01), higher importance of intimacy and sexuality (β = 0.110, *p* < .001; β = 0.094, *p* < .05), and with better health status. With regard to health status, individuals with fewer depressive symptoms (β = −0.011, *p* < .01) and a lower number of physical diseases (β = −0.033, *p* < .01) were more satisfied with their sex life, among heterosexual adults. In contrast, worse functioning (β = − 0.008, *p* < .05) was associated with lower sexual satisfaction among sexual minority adults.

In further sensitivity analysis (Supplementary Table 2), we additionally controlled for partnership assessment (variable collected only for partnered individuals) and assessment of relationship with own family. The regression analysis revealed that individuals who rated their partnership better were more satisfied with their sex life (β = −0.243, *p* < .001 among middle-aged individuals and β = −0.154, *p* < .01 among older individuals). Moreover, in another sensitivity analysis, we controlled for additional determinants of sexual satisfaction, namely income and cognitive status. In additional sensitivity analysis, we used a different definition of sexual minorities, namely, we included only individuals who identify as homosexual or bisexual. Sexual satisfaction was not associated with sexual orientation in these models either (Supplementary Tables 3 and 4).

## Discussion and Implications

### Key Results

In this large observational study based on nationally representative data, 60.1% of middle-aged individuals (aged 40–64 years) and 45.6% of older individuals (aged 65 years and above) were satisfied or very satisfied with their sex life. More importantly, the level of sexual satisfaction was similar among sexual minority and heterosexual individuals. In sum, 56% of heterosexual and 52% of sexual minority adults were satisfied or very satisfied with their sex life.

In contrary to our hypothesis, after including important individual, relationship, social support, and macrolevel variables, we found that sexual orientation was not significantly associated with sexual satisfaction both in middle-aged and older individuals. In both age groups, sexual satisfaction was associated, among other things, with lower loneliness scores and higher importance of sexuality and intimacy, and partnership satisfaction. In our analysis, for example, age, gender, and cognition were not significantly associated with sexual satisfaction. Better health status, fewer depressive symptoms, not being single, and higher general self-esteem were associated with sexual satisfaction among individuals in middle age, but not in older age.

Similar results, namely no significant differences in sexual satisfaction according to sexual orientation, were also reported in studies based on younger samples (Del Mar Sánchez-Fuentes & Sierra, 2015; Frederick et al., 2021; Kuyper & Vanwesenbeeck, 2011; Pereira et al., 2019). For example, in a large study from the Netherlands, it has been shown that sexual orientation was not significantly correlated with sexual satisfaction among younger individuals (average age 38.2 years; Kuyper & Vanwesenbeeck, 2011). Similar results were reported in the United States, based on a large convenience sample (age 18–65), namely individuals who are gay did not differ from heterosexual men with regard to sexual satisfaction (Frederick et al., 2021).

Our results are in contrast with a few previous studies. For example, a large study from England has shown that older LGB respondents (50 years and older) reported lower satisfaction with sex life than their heterosexual counterparts, both in crude analysis as well as after controlling for other factors (Grabovac et al., 2019). The evidence of sexual orientation differences in sexual satisfaction was also reported in a recent very large study in Sweden (*n* = 14,537; Björkenstam et al., 2020). According to this study, among individuals aged 16–84 years, both bisexual women and men were less satisfied with their sex life in comparison to their heterosexual counterparts (Björkenstam et al., 2020). Similar results were confirmed in a study from the United States based on a large sample with more than 4,000 participants with average age of 47 years (Flynn et al., 2017). Namely, it has been shown that heterosexual men reported higher sexual satisfaction than gay and bisexual men. However, in women, the differences were not significant (Flynn et al., 2017). In contrast, a study from Israel showed that heterosexual men have lower satisfaction with sex life than gay men (Gil, 2007). Another study based on a sample of young and middle-aged women aged 19–62 years found that lesbian/bisexual women (compared to heterosexual women) reported higher satisfaction with sex life (Henderson et al., 2009). Similar findings were reported in a sample of sexual and gender minority young adults assigned female at birth (Dyar et al., 2020).

There are many possible explanations for our quite unexpected results. Our hypothesis that sexual minority adults are on average less satisfied with their sex lives was built, among other things, on the minority stress theory (Meyer, 2003). Namely, due to the long-lasting discrimination and prejudice faced by sexual minority adults, they are, among others, at greater risk of poorer health compared to heterosexual adults, which, in turn, can negatively affect sexual satisfaction. However, it may be possible that this negative effect can be reduced by other factors. Previous research has shown that sexual satisfaction may be affected by general factors such as sociodemographic factors, availability of a partner, and relationship satisfaction rather than by LGB-specific factors (resulting from minority stress; Del Mar Sánchez-Fuentes & Sierra, 2015; Fleishman et al., 2020; Kuyper & Vanwesenbeeck, 2011). Relationship satisfaction, in particular, is frequently suggested as an important predictor of sexual satisfaction among older individuals, including those in same-sex relationships (Fleishman et al., 2020). The results regarding nonsignificant differences by sexual orientation corroborate the findings on comparable factors contributing to sexual satisfaction among both heterosexual and sexual minority adults (Frederick et al., 2021).

Further, owing to changes in attitudes toward sexual minority adults (Lyons et al., 2015) and increasing acceptance, expressing sexuality in public places is possible, irrespective of sexual orientation. In countries with a higher acceptance of same-sex sexuality, no differences in sexual satisfaction among heterosexual and non-heterosexual adults were found either (Del Mar Sánchez-Fuentes & Sierra, 2015). It has also been suggested that this social normalization may contribute to reducing internalized homonegativity and thereby increase sexual satisfaction (Del Mar Sánchez-Fuentes & Sierra, 2015; Henderson et al., 2009). Consequently, these could be the factors that contribute to greater overall sexual satisfaction, especially among sexual minority adults.

Accordingly, it is possible that for these reasons, we did not find differences in the levels of sexual satisfaction between sexual minority and heterosexual adults in our analysis. In addition, older sexual minority adults may have good coping strategies for adverse events (e.g., stigmatization, discrimination), which further may reduce the disparities in sexual satisfaction in comparison to heterosexual adults.

The differences between the results of our study and those of the recent literature could also be attributable to the sampling strategy. A large number of studies were based on rather small, nonrepresentative samples. In addition, many samples were relatively young. It is possible that differences in sexual satisfaction are more evident in younger cohorts and reduce in middle-aged and older individuals. This should be further explored in longitudinal studies.

### Strengths and Limitations

The strengths of our study include the use of a large, nationally representative sample. Moreover, we included both heterosexual individuals and sexual minority adults. This is one of the first studies analyzing sexual satisfaction with regard to sexual orientation also among older adults. The presented data are cross-sectional and it is therefore difficult to draw causal conclusions. Further research based on longitudinal data is needed. The question about sexual orientation and sexual satisfaction was placed in the drop-off questionnaire. About 73% of individuals filled out this additional questionnaire. For this reason, the analytical sample was smaller than the full sample. In order to account for the panel attrition, in all analyses, we used drop-off sampling weights. In addition, the missing values were addressed in sensitivity analysis using the FIML. In terms of significance, the results of the analysis remained virtually the same. Our analyses are based on data collected in 2008. However, this was the only wave in which data on both sexual orientation and sexual satisfaction were gathered. In our sample, we did not have additional variables that measured the number of sexual activities. However, the number of sexual activities is not always the most important factor contributing to sexual satisfaction. For example, relationship satisfaction also seems to be highly relevant (Lee et al., 2016). Further, in our analysis, we controlled for family status as well as for the assessment of relationship. It should be noted that most effect sizes in the regression analysis were rather small. A single-item measure was used to quantify sexual satisfaction. It is a global measure that reflects the overall assessment of sexual satisfaction. The single-item measure has been shown to have convergent validity with other scales that measure sexual satisfaction (Global Measure of Sexual Satisfaction and the New Sexual Satisfaction Scale—Short; Mark et al., 2014). Due to its economy compared to other scales, such a single-item measure is often used in large studies (Mark et al., 2014). However, in clinical settings, other scales that include more dimensions may be more appropriate.

## Conclusion

Our analysis showed that sexual orientation was not significantly associated with sexual satisfaction among both middle-aged and older adults. In contrast, in both age groups, higher sexual satisfaction was associated with, among others, lower loneliness and higher importance of sexuality and intimacy, and partnership satisfaction. For individuals in middle age, better health status, fewer depressive symptoms, not being single (availability of partner), and higher general self-esteem were associated with higher sexual satisfaction. One may conclude that these factors particularly need to be taken into account when investigating sexual satisfaction. The relationship between sexual orientation and sexual satisfaction should also be studied among older people in other countries.

In our analyses, we showed that satisfaction with sex life declined in a similar way with age among both heterosexual and sexual minority adults. Approximately 45% of older individuals (aged 65 years and above), regardless of their sexual orientation, were satisfied with their sex life. Our results emphasize strong similarities in the level of satisfaction with sex life and the changes in this satisfaction with age among heterosexual individuals and sexual minority adults.

## Supplementary Material

igad010_suppl_Supplementary_Material
