# Supplementary material for: Sexual Satisfaction Among Sexual Minority and Heterosexual Middle-Aged and Older Adults
Source: Innov Aging. 2023 Jan 31;7(2):igad010. doi: 10.1093/geroni/igad010 (PMC10053635; doi:10.1093/geroni/igad010)
Supplement: igad010_suppl_Supplementary_Material [file igad010_suppl_supplementary_material.pdf]

Innovation in Aging Online Supplementary Material**:** Elżbieta W. Buczak-Stec, Hans-Helmut König, & André Hajek. Sexual satisfaction among sexual minority and heterosexual middle-aged and older adults.

Table S1 Sensitivity analysis. Results of multiple regression analysis. Sexual satisfaction among heterosexual and sexual minority adults.

**Variable**	**Heterosexual adults**		**Sexual minority adults**	
	**Coef.**	**SE**	**Coef.**	**SE**
Individual level				
Female (ref. male)	0.002	(0.038)	0.231	(0.134)
Age	0.000	(0.003)	-0.009	(0.009)
Level of education (ref. low)				
Medium	-0.011	(0.072)	0.026	(0.162)
High	-0.084	(0.076)	0.098	(0.186)
Type of district (ref. large cities)				
Urban cities	0.032	(0.044)	-0.021	(0.140)
Urban-rural districts	0.126*	(0.051)	-0.004	(0.149)
Rural districts	0.065	(0.055)	-0.226	(0.226)
Labour force status (ref. working)				
Retired	-0.040	(0.058)	-0.084	(0.183)
Not employed	-0.001	(0.060)	-0.191	(0.172)
Physical functioning (from 0 = worst score to 100 = best score)	-0.002	(0.001)	-0.008*	(0.003)
Total number of physical diseases	-0.033**	(0.012)	-0.027	(0.034)
Self-rated health (from 1 = very good to 5 = very bad)	-0.047	(0.026)	0.083	(0.092)
Depressive symptoms	-0.011**	(0.004)	-0.011	(0.012)
Self-esteem	0.107	(0.056)	0.219	(0.153)
Smoking habits (ref. never)				
Used to	-0.053	(0.039)	-0.175	(0.128)
Stopped smoking	-0.364	(0.201)	-0.510	(0.394)
Occasionally	-0.008	(0.098)	-0.589	(0.300)
Daily	-0.081	(0.055)	-0.601**	(0.200)
Physical activity (ref. daily)				
Daily	0.076	(0.079)	0.124	(0.167)
Several times a week	0.031	(0.081)	-0.298	(0.189)
Once a week	0.065	(0.091)	-0.134	(0.240)
Between 1-3 times per month	0.099	(0.088)	-0.329	(0.231)
Less often	0.070	(0.080)	-0.191	(0.160)
Importance of sexuality and intimacy (from 1 = lowest to 7 = highest):	0.110***	(0.012)	0.094*	(0.039)
Partnership level				
Type of partnership (ref. married)				
Divorced	-0.053	(0.065)	-0.572*	(0.231)
Widowed	-0.056	(0.059)	0.063	(0.172)
Single	-0.286***	(0.073)	-0.211	(0.214)
Social support variable				
Network size (number of important persons with whom you have with regular contact	-0.005	(0.006)	-0.040	(0.022)
Loneliness (from 1 = very low to 4 = very high)	-0.250***	(0.038)	-0.358**	(0.112)
Macrolevel				
Church visits (ref. several times a week or once a week)				
1-3 times per month / Several times a year	-0.042	(0.063)	0.185	(0.207)
Less often / never	-0.061	(0.061)	0.035	(0.195)
Constant	3.432***	(0.338)	4.551***	(1.139)
Observations	4,483		373	
R-squared	0.125		0.288	

Note: Coef. - estimated coefficients; SE - robust standard errors in parentheses; Cross-sectional drop-off weights were included in regression models; Education level according to ISCED (Matthews et al.). Type of district according to Federal Institute for Research on Building, Urban Affairs and Spatial Development. Physical functioning was measured by the subscale “Physical Functioning” of SF-36 Short Form Health Survey (0-100 range) (Ware Jr & Sherbourne, 1992). The Center for Epidemiological Studies Depression Scale (CES-D) was used to quantify depressive symptoms (Radloff, 1977). Self-esteem - Rosenberg scale; range 1-4, high values representing high self-esteem (Ferring & Filipp, 1996). Loneliness was assessed using the De Jong Gierveld Loneliness scale.
*** $p<0.001$, ** $p<0.01$, * $p<0.05$

Table S2 Sensitivity analysis. Results of multiple regression analysis. Sexual satisfaction and sexual orientation among middle-aged (40-64 years old) and older (65 years and above) individuals. Additionally, controlled for partnership assessment (variable collected only for partnered individuals) and assessment of relationship with own family.

**Variable**	**Total sample**		**Middle-aged adults (40-64 years)**		**Older adults (65 years and above)**	
	**Coef.**	**SE**	**Coef.**	**SE**	**Coef.**	**SE**
Individual level						
Sexual orientation (ref. heterosexual)						
Sexual minority adults	0.077	(0.079)	0.068	(0.120)	0.061	(0.087)
Female (ref. male)	0.021	(0.042)	-0.015	(0.051)	0.108	(0.059)
Age	-0.001	(0.003)	-0.000	(0.004)	-0.003	(0.005)
Level of education (ref. low)						
Medium	0.005	(0.079)	0.123	(0.131)	-0.097	(0.078)
High	-0.075	(0.082)	0.022	(0.134)	-0.127	(0.082)
Type of district (ref. large cities)						
Urban cities	0.018	(0.047)	0.013	(0.063)	0.023	(0.061)
Urban-rural districts	0.104	(0.054)	0.108	(0.070)	0.067	(0.070)
Rural districts	0.090	(0.059)	0.096	(0.080)	0.075	(0.069)
Labour force status (ref. working)						
Retired	-0.065	(0.061)	-0.074	(0.080)	0.030	(0.202)
Not employed	0.046	(0.065)	0.064	(0.070)	0.131	(0.223)
Physical functioning (from 0 = worst score to 100 = best score)	-0.001	(0.001)	-0.002	(0.002)	0.002	(0.001)
Total number of physical diseases	-0.038**	(0.012)	-0.043*	(0.017)	-0.033*	(0.016)
Self-rated health (from 1 = very good to 5 = very bad)	-0.026	(0.028)	-0.038	(0.036)	0.007	(0.040)
Depressive symptoms	-0.007	(0.004)	-0.007	(0.005)	-0.006	(0.007)
Self-esteem	0.147*	(0.061)	0.193*	(0.077)	0.013	(0.088)
Smoking habits (ref. never)						
Used to	-0.078	(0.040)	-0.061	(0.053)	-0.081	(0.054)
Stopped smoking	-0.396	(0.214)	-0.333	(0.236)	-0.668	(0.461)
Occasionally	-0.045	(0.096)	-0.041	(0.107)	-0.021	(0.199)
Daily	-0.096	(0.061)	-0.095	(0.070)	-0.015	(0.116)
Physical activity (ref. daily)						
Daily	0.090	(0.082)	0.080	(0.110)	0.078	(0.106)
Several times a week	0.016	(0.085)	-0.029	(0.113)	0.095	(0.112)
Once a week	0.039	(0.094)	0.019	(0.122)	0.069	(0.134)
Between 1-3 times per month	0.036	(0.092)	-0.013	(0.121)	0.154	(0.120)
Less often	0.028	(0.085)	-0.037	(0.116)	0.159	(0.106)
Importance of sexuality and intimacy (from 1 = lowest to 7 = highest):	0.077***	(0.015)	0.059**	(0.021)	0.111***	(0.017)
Partnership level						
Type of partnership (ref. married)						
Divorced	0.226*	(0.089)	0.233*	(0.097)	0.212	(0.195)
Widowed	0.078	(0.118)	0.160	(0.149)	-0.012	(0.178)
Single	-0.309*	(0.136)	-0.311*	(0.149)	-0.163	(0.255)
Assessment of partnership (1=very good to 5=very bad)	-0.218***	(0.032)	-0.243***	(0.041)	-0.154**	(0.048)
Assessment of relationship with own family (1=very good to 5=very bad)	0.036	(0.025)	0.061	(0.032)	-0.025	(0.038)
Social support variables						
Network size (number of important persons with whom you have with regular contact	-0.008	(0.007)	-0.008	(0.009)	-0.004	(0.008)
Loneliness (from 1 = very low to 4 = very high)	-0.186***	(0.043)	-0.175***	(0.053)	-0.222**	(0.068)
Macrolevel						
Church visits (ref. several times a week or once a week)						
1-3 times per month / Several times a year	-0.021	(0.067)	0.051	(0.100)	-0.132	(0.073)
Less often / never	-0.051	(0.065)	-0.016	(0.097)	-0.087	(0.071)
Constant	3.529***	(0.327)	3.480***	(0.423)	3.578***	(0.588)
Observations	3,951		2,337		1,614	
R-squared	0.115		0.112		0.144	

Note: Coef. - estimated coefficients; SE - robust standard errors in parentheses; Cross-sectional drop-off weights were included in regression models; Education level according to ISCED (Matthews et al.). Type of district according to Federal Institute for Research on Building, Urban Affairs and Spatial Development. Physical functioning was measured by the subscale "Physical Functioning" of SF-36 Short Form Health Survey (0-100 range) (Ware Jr & Sherbourne, 1992). The Center for Epidemiological Studies Depression Scale (CES-D) was used to quantify depressive symptoms (Radloff, 1977). Self-esteem - Rosenberg scale; range 1-4, high values representing high self-esteem (Ferring & Filipp, 1996). Loneliness was assessed using the De Jong Gierveld Loneliness scale.

*** p<0.001, ** p<0.01, * p<0.05

Table S3 Sensitivity analysis. Results of multiple regression analysis. Sexual satisfaction and sexual orientation among middle-aged (40-64 years old) and older (65 years and above) individuals. Additionally, controlled for income and cognitive status.

**Variable**	**Total sample**		**Middle-aged adults (40-64 years)**		**Older adults (65 years and above)**	
	**Coef.**	**SE**	**Coef.**	**SE**	**Coef.**	**SE**
Individual level						
Sexual orientation (ref. heterosexual)						
Sexual minority adults	0.059	(0.091)	0.026	(0.134)	0.073	(0.096)
Female (ref. male)	0.037	(0.048)	0.003	(0.059)	0.119	(0.069)
Age	-0.000	(0.003)	0.001	(0.005)	-0.005	(0.006)
Level of education (ref. low)						
Medium	-0.003	(0.097)	0.188	(0.154)	-0.215*	(0.093)
High	-0.086	(0.102)	0.073	(0.158)	-0.200*	(0.098)
Type of district (ref. large cities)						
Urban cities	-0.054	(0.052)	-0.095	(0.067)	0.042	(0.074)
Urban-rural districts	0.045	(0.058)	0.042	(0.073)	0.007	(0.083)
Rural districts	0.030	(0.068)	-0.006	(0.089)	0.119	(0.086)
Labour force status (ref. working)						
Retired	-0.054	(0.068)	-0.099	(0.088)	-0.132	(0.274)
Not employed	0.113	(0.066)	0.136	(0.071)	-0.003	(0.295)
Physical functioning (from 0 = worst score to 100 = best score)	-0.002	(0.001)	-0.003	(0.002)	0.001	(0.002)
Total number of physical diseases	-0.027	(0.014)	-0.023	(0.020)	-0.040*	(0.019)
Self-rated health (from 1 = very good to 5 = very bad)	-0.035	(0.033)	-0.050	(0.041)	-0.004	(0.047)
Depressive symptoms	-0.005	(0.005)	-0.004	(0.006)	-0.005	(0.008)
Self-esteem	0.188**	(0.071)	0.239**	(0.088)	-0.009	(0.097)
Smoking habits (ref. never)						
Used to	-0.077	(0.047)	-0.074	(0.061)	-0.068	(0.063)
Stopped smoking	-0.313	(0.265)	-0.346	(0.290)	-0.092	(0.420)
Occasionally	0.028	(0.101)	0.037	(0.114)	-0.002	(0.191)
Daily	-0.092	(0.071)	-0.085	(0.080)	-0.090	(0.148)
Physical activity (ref. daily)						
Daily	-0.005	(0.091)	-0.128	(0.118)	0.192	(0.124)
Several times a week	-0.044	(0.094)	-0.191	(0.123)	0.212	(0.126)
Once a week	-0.044	(0.106)	-0.161	(0.133)	0.084	(0.159)
Between 1-3 times per month	-0.015	(0.100)	-0.152	(0.128)	0.247	(0.132)
Less often	-0.009	(0.092)	-0.177	(0.123)	0.287*	(0.121)
Importance of sexuality and intimacy (from 1 = lowest to 7 = highest):	0.085***	(0.018)	0.075**	(0.025)	0.116***	(0.020)
Partnership level						
Type of partnership (ref. married)						
Divorced	0.206*	(0.103)	0.235*	(0.111)	0.033	(0.221)
Widowed	-0.020	(0.147)	0.105	(0.178)	-0.221	(0.240)
Single	-0.366*	(0.155)	-0.378*	(0.165)	-0.118	(0.349)
Assessment of partnership (1=very good to 5=very bad)	-0.212***	(0.037)	-0.244***	(0.045)	-0.117*	(0.058)
Assessment of relationship with own family (1=very good to 5=very bad)	0.036	(0.030)	0.050	(0.036)	-0.003	(0.044)
Social support variables						
Network size (number of important persons with whom you have with regular contact	-0.008	(0.008)	-0.011	(0.010)	0.003	(0.009)
Loneliness (from 1 = very low to 4 = very high)	-0.192***	(0.050)	-0.177**	(0.061)	-0.264**	(0.080)
Macrolevel						
Church visits (ref. several times a week or once a week)						
1-3 times per month / Several times a year	0.031	(0.079)	0.074	(0.115)	-0.047	(0.090)
Less often / never	-0.031	(0.078)	-0.023	(0.112)	-0.019	(0.090)
Income (logged)	0.021	(0.045)	0.004	(0.057)	0.044	(0.068)
Cognitive functioning	0.000	(0.002)	0.001	(0.002)	-0.003	(0.002)
Constant	3.358***	(0.574)	3.432***	(0.716)	3.868***	(0.930)
Observations	2,967		1,812		1,155	
R-squared	0.111		0.116		0.143	

Note: Coef. - estimated coefficients; SE - robust standard errors in parentheses; Cross-sectional drop-off weights were included in regression models; Education level according to ISCED (Matthews et al.). Income - monthly equivalence income (new OECD equivalence scale). Type of district according to Federal Institute for Research on Building, Urban Affairs and Spatial Development. Physical functioning was measured by the subscale "Physical Functioning" of SF-36 Short Form Health Survey (0-100 range) (Ware Jr & Sherbourne, 1992). The Center for Epidemiological Studies Depression Scale (CES-D) was used to quantify depressive symptoms (Radloff, 1977). Cognitive functioning was assessed with help of adaptation of the Digit Symbol Substitution Test; range 1-92, higher values indicate better cognitive functioning. Self-esteem - Rosenberg scale;

range 1-4, high values representing high self-esteem (Ferring & Filipp, 1996). Loneliness was assessed using the De Jong Gierveld Loneliness scale.
*** $p<0.001$, ** $p<0.01$, * $p<0.05$

Table S4 Sensitivity analysis. Results of multiple regression analysis. Sexual satisfaction and sexual orientation among middle-aged (40-64 years old) and older (65 years and above) individuals. Different definition of sexual minorities - sexual orientation (heterosexual; sexual minority adults [homosexual, bisexual]).

**Variable**	**Total sample**		**Middle-aged adults (40-64 years)**		**Older adults (65 years and above)**	
	**Coef.**	**SE**	**Coef.**	**SE**	**Coef.**	**SE**
Individual level						
Sexual orientation (ref. heterosexual)						
Sexual minority adults (homosexual, bisexual)	-0.006	(0.122)	0.030	(0.201)	-0.086	(0.118)
Female (ref. male)	0.030	(0.049)	-0.004	(0.060)	0.116	(0.071)
Age	-0.000	(0.003)	0.001	(0.005)	-0.005	(0.006)
Level of education (ref. low)						
Medium	-0.005	(0.101)	0.189	(0.158)	-0.239*	(0.095)
High	-0.079	(0.106)	0.085	(0.162)	-0.217*	(0.100)
Type of district (ref. large cities)						
Urban cities	-0.053	(0.052)	-0.076	(0.068)	0.015	(0.075)
Urban-rural districts	0.042	(0.059)	0.045	(0.074)	0.018	(0.087)
Rural districts	0.027	(0.069)	-0.003	(0.089)	0.125	(0.090)
Labour force status (ref. working)						
Retired	-0.051	(0.070)	-0.103	(0.090)	-0.120	(0.261)
Not employed	0.134*	(0.068)	0.152*	(0.073)	0.020	(0.284)
Physical functioning (from 0 = worst score to 100 = best score)	-0.001	(0.001)	-0.003	(0.002)	0.001	(0.002)
Total number of physical diseases	-0.028	(0.015)	-0.024	(0.020)	-0.038	(0.020)
Self-rated health (from 1 = very good to 5 = very bad)	-0.032	(0.034)	-0.051	(0.041)	0.005	(0.048)
Depressive symptoms	-0.007	(0.005)	-0.005	(0.006)	-0.012	(0.006)
Self-esteem	0.172*	(0.071)	0.218*	(0.088)	-0.011	(0.095)
Smoking habits (ref. never)						
Used to	-0.068	(0.048)	-0.070	(0.063)	-0.055	(0.065)
Stopped smoking	-0.335	(0.272)	-0.389	(0.295)	-0.038	(0.458)
Occasionally	0.044	(0.105)	0.029	(0.116)	0.167	(0.203)
Daily	-0.084	(0.072)	-0.082	(0.080)	-0.042	(0.156)
Physical activity (ref. daily)						
Daily	-0.030	(0.093)	-0.157	(0.119)	0.188	(0.130)
Several times a week	-0.055	(0.097)	-0.202	(0.125)	0.215	(0.132)
Once a week	-0.042	(0.109)	-0.159	(0.135)	0.104	(0.169)
Between 1-3 times per month	-0.017	(0.102)	-0.153	(0.130)	0.231	(0.135)
Less often	-0.013	(0.095)	-0.166	(0.125)	0.262*	(0.127)
Importance of sexuality and intimacy (from 1=lowest to 7=highest):	0.090***	(0.018)	0.082***	(0.025)	0.114***	(0.021)
Partnership level						
Type of partnership (ref. married)						
Divorced	0.281**	(0.092)	0.313**	(0.100)	0.044	(0.229)
Widowed	-0.037	(0.154)	0.106	(0.181)	-0.271	(0.257)
Single	-0.362*	(0.156)	-0.379*	(0.164)	-0.041	(0.344)
Assessment of partnership (1=very good to 5=very bad)	-0.222***	(0.038)	-0.253***	(0.046)	-0.132*	(0.058)
Assessment of relationship with own family (1=very good to 5=very bad)	0.031	(0.031)	0.044	(0.038)	-0.007	(0.046)
Social support variables						
Network size (number of important persons with whom you have with regular contact	-0.005	(0.008)	-0.007	(0.010)	0.004	(0.010)
Loneliness (from 1 = very low to 4 = very high)	-0.188***	(0.050)	-0.180**	(0.062)	-0.237***	(0.067)
Macrolevel						
Church visits (ref. several times a week or once a week)						
1-3 times per month / Several times a year	0.037	(0.081)	0.088	(0.117)	-0.045	(0.093)
Less often / never	-0.027	(0.080)	-0.003	(0.114)	-0.038	(0.091)
Income (logged)	0.004	(0.045)	-0.011	(0.057)	0.032	(0.069)
Cognitive functioning	0.000	(0.002)	0.001	(0.002)	-0.002	(0.002)
Constant	3.498***	(0.576)	3.599***	(0.721)	3.943***	(0.955)
Observations	2,848		1,753		1,095	
R-squared	0.115		0.119		0.150	

Note: Coef. - estimated coefficients; SE - robust standard errors in parentheses; Cross-sectional drop-off weights were included in regression models; Education level according to ISCED (Matthews et al.). Income - monthly equivalence income (new OECD equivalence scale). Type of district according to Federal Institute for Research on Building, Urban Affairs and Spatial Development. Physical functioning was measured by the subscale "Physical Functioning" of SF-36 Short Form Health Survey (0-100 range) (Ware Jr & Sherbourne, 1992). The Center for Epidemiological Studies Depression Scale (CES-D) was used to quantify depressive symptoms (Radloff, 1977). Cognitive functioning was assessed with help of adaptation of the Digit Symbol Substitution Test; range 1-92, higher values indicate better cognitive functioning. Self-esteem - Rosenberg scale; range 1-4, high values representing high self-esteem (Ferring & Filipp, 1996). Loneliness was assessed using the De Jong Gierveld Loneliness scale. *** p<0.001, ** p<0.01, * p<0.05
